# Patellar Tendon Avulsion with Tibial Tuberosity Sleeve Fragment

**DOI:** 10.1155/2019/6193498

**Published:** 2019-03-12

**Authors:** Akira Isaka, Satoshi Ichihara, Yasuhiro Homma, Tomohiko Hirose, Hajime Kajihara

**Affiliations:** ^1^Hirose Hospital, Kagawa 760-0079, Japan; ^2^Hand Surgery Center, Juntendo University Urayasu Hospital, Chiba 279-0021, Japan; ^3^Department of Orthopedic Surgery, Juntendo University, Tokyo 113-8431, Japan; ^4^Department of Orthopedic Surgery, Koto Hospital, Tokyo 136-0072, Japan

## Abstract

Rupture of the patellar tendon is relatively rare. We report a case of patellar tendon avulsion with a tibial tuberosity sleeve fragment in pediatric patient. In pediatric patient, diagnosis is sometimes difficult due to uncompleted ossification. In the present case, which involved the presence of a small fleck of bone from tibia, we were able to attain a diagnosis using the Koshino-Sugimoto index and MRI and easily determine the optimal treatment with the use of the suture anchor and tension band wiring method.

## 1. Introduction

Rupture of the patellar tendon is relatively rare. The rates of patellar tendon injury is 3% of all knee extension system injuries [1]. In most cases, the patellar tendon is ruptured from the upper end as a sleeve fracture of the patella [2, 3]. We herein report a case of patellar tendon avulsion with a tibial tuberosity sleeve fragment in pediatric patient and discuss the diagnosis and treatment with a brief review of the literatures.

## 2. Case Presentation

**Figure 1:**
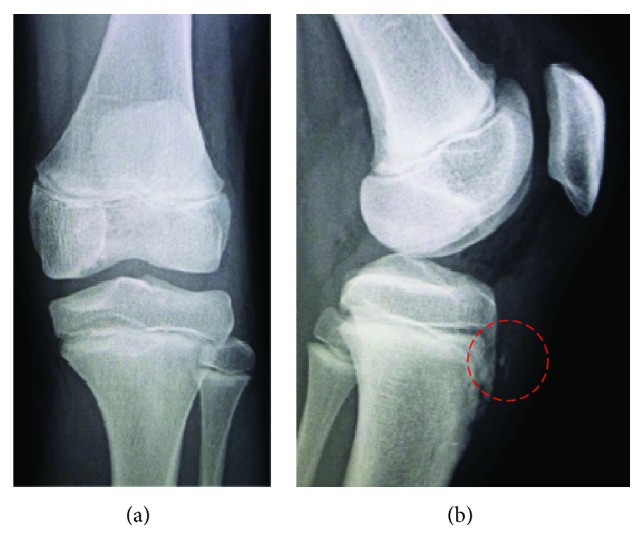
X-rays and computed tomography (CT) showed small flecks of bone at proximal tibial tuberosity.

**Figure 2:**
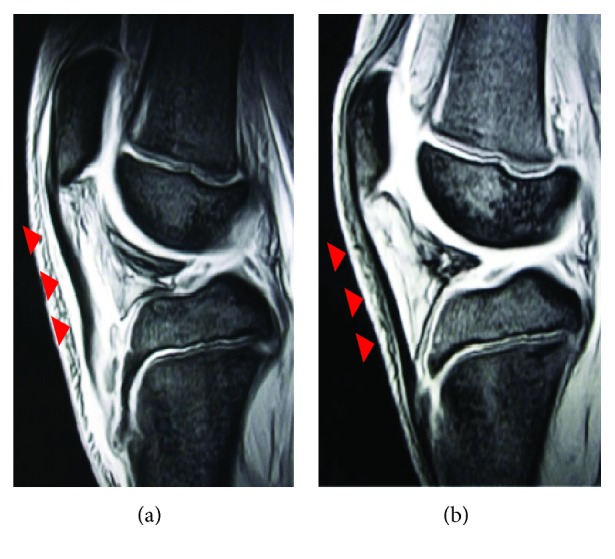
(a) Left is affected side. (b) Right is healthy side. Magnetic resonance imaging (MRI) revealed swelling and loosening of the patellar tendon.

**Figure 3:**
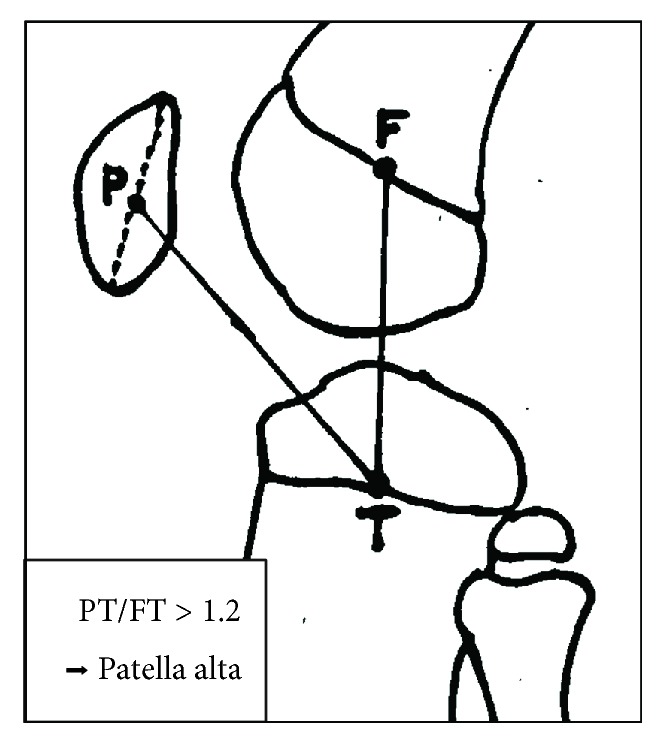
Koshino-Sugimoto index.

**Figure 4:**
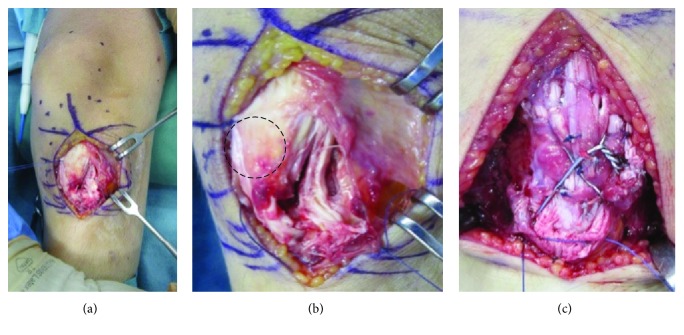
Operative findings. The patellar tendon was avulsed with sleeve fragment from tibial tuberosity.

**Figure 5:**
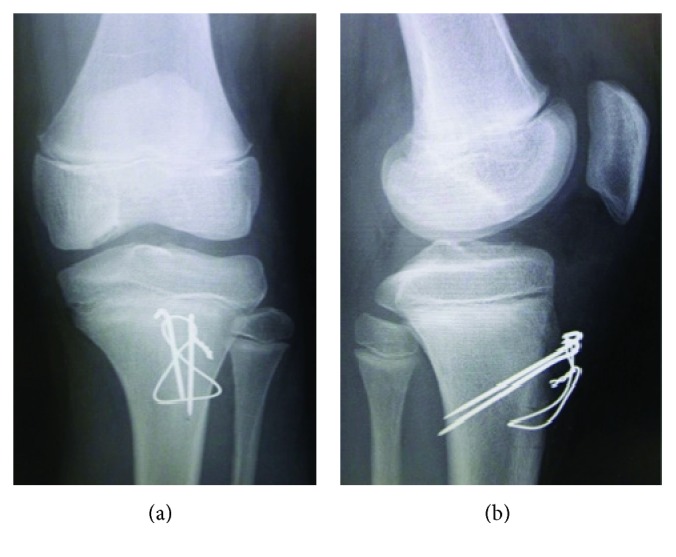
The avulsed tendon was repaired using two suture anchors, and fragment including cartilage was attached by tension band wiring method.

A 12-year-old boy was referred to our hospital with an acute injury of the left knee that had occurred while playing soccer. Slight swelling and pain at the knee were observed. Assessment of active range of motion was impossible due to pain. The patient had no history of chronic medication or Osgood-Schlatter disease. Radiographs and computed tomography (CT) showed small flecks of bone at the proximal tibial tuberosity (Figure 1). Magnetic resonance imaging (MRI) revealed swelling and loosening of the patellar tendon (Figure 2). We also recognized patella alta in comparison with the right knee with a Sugimoto index of 1.36 [4] (Figure 3). Based on these clinical and radiological findings, we diagnosed patellar tendon avulsion with a tibial tuberosity sleeve fragment and determined that surgery was required. Surgery was performed 9 days after the injury. During the operation, we found that the patellar tendon was avulsed with a sleeve fragment from the tibial tuberosity (Figure 4). The avulsed tendon was repaired using two suture anchors, and the fragment including cartilage was attached by the tension band wiring method (Figure 5). After the fixation, the height of the left patella was fluoroscopically confirmed to be at the same level as the right patella. The knee was immobilized by casting with a slightly flexed knee position for 3 weeks. After 3 weeks, rehabilitation of active and passive range of motion was started. The K-wire and soft wire were removed 4 months after the surgery. At 6 months postoperatively, active range of motion of the knee was equal to that of the contralateral side without pain. At the 1-year follow-up, no complication was observed.

## 3. Discussion

The main cause of extensor mechanism injuries of the knee is forceful contraction of the quadriceps against a partially flexed knee or firm resistance. The quadriceps undergoes an eccentric load avulsing the patellar tendon complex. Failure of the patellar tendon is relatively uncommon among extensor mechanism injuries of the knee [1, 5]. Zernicke et al. [6] reported that a load of 17.5 times the body weight is required to rupture the human patellar tendon. In most cases of patellar tendon rupture, the patient has tendon degeneration due to chronic renal failure or diabetes. In pediatric patients with patellar tendon avulsion at the distal attachment of the tibia with small fragments due to uncompleted ossification, such as the patient described herein, the diagnosis is difficult and the gold standard treatment has not yet been established.

As a disorder or injury that we should distinguish from our case, Osgood-Schlatter syndrome (OSS) and avulsion fracture of tibial tuberosity are common. OSS suffer the tibial tuberosity in growing children and presents with local pain, swelling, and tenderness of the tuberosity. X-ray shows fleck of bone at tibial tuberosity. However, it is not a traumatic disease but disorder like enthesopathy. Our case existed the history of trauma and no pain before injury. Moreover, while OSS does not show the obvious patella alta on X-ray, our case shows the obvious patella alta on X-ray. Of course, it is unclear whether OSS is one of the risk factor for our case. Our case may have the possibility of vulnerability cause of immature bone or asymptomatic OSS.

Whereas the distinguishing between patella tendon rupture and avulsion fracture was difficult in the present case because the fleck of bone was tiny and the displacement was minimal. Desai and Parikh [5] reported that a high-riding patella, palpable gap at the site of the patellar tendon, and an inability to perform active extension are important findings suggestive of patellar tendon injuries. Although physical examination is important, such examination is difficult to perform because of the swelling and pain around the knee in pediatric patients. MRI and fluoroscopy are useful for comparison with the opposite knee. MRI shows loosening of the patellar tendon (Figure 2), and the patellar height is easily comparable. Kosuge et al. [7] described the feasibility of ultrasonography as a noninvasive examination in the diagnosis of these fractures. If obvious patella alta is present, the diagnosis is easily made. However, the patella alta was not obvious in our case. The Insall-Salvati index cannot be applied in pediatric patients because of the growth plate; however, patella alta can be assessed with the Koshino-Sugimoto index [4]. This index is useful in pediatric patients with a remaining growth plate. With respect to treatment, it is important to attach the tendon by a suture anchor or the pull-out method because the bone fragment is small. The fragment is not large enough for screw fixation, and the suture anchor and tension band wiring method were required in our case. In a previous study, although cerclage wiring was used as augmentation with reattachment of the tendon or fragment, skin irritation often occurs when such wiring is used [7]. The tendon injury itself may need augmentation with an allograft or cerclage wiring [8].

In conclusion, when a pediatric patient sustains rupture of the patellar tendon from the tibial attachment, diagnosis is sometimes difficult due to uncompleted ossification. In the present case, which involved the presence of a small fleck of bone from tibia, we were able to attain a diagnosis using the Koshino-Sugimoto index and MRI and easily determine the optimal treatment.
